# EntroMEMS: Proof-of-Concept Integration of Quantum-Derived Initialization and MEMS Entropy Acquisition on an ESP32

**DOI:** 10.3390/s26206494

**Published:** 2026-10-14

**Authors:** Borislav Atanasov Georgiev

**Affiliations:** 1 Department of Machine and Precision Engineering, Technical University of Gabrovo, 4 H. Dimitar Str., 5300 Gabrovo, Bulgaria; gborislav@gbg.bg; Tel.: +359-898-518-724; 2 Center of Competence “Smart Mechatronic, Eco- and Energy-Saving Systems and Technologies”, Technical University of Gabrovo, 5300 Gabrovo, Bulgaria

**Keywords:** Quantum Random Number Generator (QRNG), hybrid quantum-classical security, MEMS sensors, entropy conditioning, Industrial Internet of Things (IIoT), embedded security

## Abstract

**Highlights:**

**What are the main findings?**

- A proof-of-concept cloud-to-edge architecture was implemented, combining a 1024-bit quantum-derived initialization value from an IBM Quantum backend with structured acquisition of candidate physical entropy from three MEMS gyroscopes on an ESP32 platform.
- The revised evaluation includes an official NIST SP 800-22 STS 2.1.2 single-sequence assessment of a contiguous 1,000,000-bit conditioned sequence and official NIST SP 800-90B v1.1.8 non-IID sequential assessments of V2 raw binary samples; the lowest observed non-IID estimate was 0.815998 bits per sample.

**What are the implications of the main findings?**

- The measurements support the feasibility of EntroMEMS as a laboratory prototype and its potential use as an auxiliary input for entropy diversification; they do not establish a protected root of trust or production cryptographic security.
- The revised study quantifies acquisition throughput, memory footprint, cross-run stability, and behavior under selected orientation, perturbation, and moderate-heating conditions while explicitly identifying the absence of energy and provisioning-latency measurements and restart validation as limitations.

**Abstract:**

Resource-constrained edge devices may have limited entropy during startup. EntroMEMS is a proof-of-concept architecture combining quantum-derived initialization material with candidate physical entropy from three microelectromechanical systems (MEMS) gyroscopes on an ESP32. A 1024-bit quantum-derived pool was embedded in firmware; its first 128 bits initialize the prototype generator. This does not constitute secure provisioning. The revised V2 acquisition retains two least significant bits from each gyroscope axis, yielding 18 binary samples per cycle. Sequential non-IID assessments using the official NIST SP 800-90B software yielded estimates of 0.825393–0.911960 bit/sample across nine baseline sensor streams and 0.815998–0.912364 bit/sample across sensitivity windows. Full SP 800-90B validation was not performed because the prescribed restart dataset was not collected. For a historical V1 conditioned sequence of 1,000,000 bits, all 188 *p*-values from the official NIST SP 800-22 suite were at least 0.01. Only one sequence was evaluated; across-sequence pass proportions and *p*-value uniformity were not assessed. A follow-up benchmark averaged 1414.287 microseconds per acquisition-and-extraction cycle, corresponding to 12.7273 kbit/s excluding serial output. These results support prototype feasibility and investigation of an auxiliary entropy input, without establishing cryptographic suitability. Production deployment would require secure provisioning, protected storage, a vetted DRBG, continuous health tests, and conservative entropy accounting.

## 1. Introduction

Secure communication protocols ultimately depend on correctly generated keys, nonces, and initialization values, yet ensuring entropy availability during early startup remains challenging on some resource-constrained platforms [1,2].

The rapid expansion of the industrial Internet of Things (IIoT) and cyber-physical systems has increased reliance on resource-constrained edge devices [3,4,5]. These platforms perform sensing and autonomous processing while also supporting authentication, protected communication, and integrity functions. Their security therefore depends on both sound cryptographic design and correctly managed entropy, keys, and device identities.

Modern cryptographic protocols ultimately rely on the availability of high-quality entropy for secure key generation, nonce creation, and cryptographic initialization [1,2]. However, embedded microcontrollers often possess only limited hardware entropy sources, and deterministic software initialization may expose devices to predictable random number generation during system startup. This limitation is particularly relevant in Industrial IoT deployments, where long operational lifetimes, unattended installations, and constrained computational resources require lightweight yet trustworthy security mechanisms.

The practical consequences of weak initialization are well documented. Heninger et al. found widespread repeated or factor-sharing public keys in deployed TLS and SSH systems and linked these failures to insufficient randomness during key generation [6]. This evidence motivates conservative entropy accounting and explicit separation between prototype statistics and cryptographic assurance.

Recent advances in quantum computing have enabled remote access to genuine quantum measurement outcomes through cloud-based quantum computing services [7,8,9]. Quantum measurement outcomes arise from fundamentally probabilistic physical processes, whereas pseudorandom generators produce outputs deterministically. Quantum measurements therefore represent an attractive source of initialization entropy. Although quantum random number generation has been extensively investigated, directly integrating quantum-generated entropy into low-cost embedded edge devices [10,11,12,13,14,15] remains challenging due to hardware accessibility, communication latency, and practical deployment constraints.

Embedded sensing platforms contain physical components whose digitized outputs may contain stochastic variation. Prior work has studied accelerometers as randomness sources and has shown that sensor-based entropy must be evaluated under explicit operating conditions and with dependency-aware estimators [16,17,18]. A recent study also investigated entropy extraction from commercial smartwatch sensors under still and shake acquisition modes [19]. Sensor availability alone does not establish cryptographic entropy: marginal balance, temporal dependence, environmental sensitivity, conditioning, and source-health behavior must be evaluated separately. EntroMEMS therefore treats the gyroscope output as candidate physical entropy and reports both descriptive statistics and official non-IID sequential estimates without claiming complete entropy-source validation.

This work presents EntroMEMS as a proof-of-concept cloud-to-edge architecture combining quantum-derived initialization material with locally acquired MEMS samples. The contributions of the revised study include a reproducible V2 acquisition model, dependency-aware non-IID estimation, an official single-sequence SP 800-22 assessment of conditioned output, stability experiments, and embedded-platform benchmarking. The study deliberately separates observed statistical behavior from production security mechanisms that were not implemented.

The main contributions of this paper are summarized as follows:

- A proof-of-concept cloud-to-edge architecture that combines quantum-derived initialization with locally acquired candidate MEMS entropy.
- An experimental workflow for provisioning an ESP32 edge platform using a 1024-bit seed generated on a real IBM Quantum backend.
- A multi-sensor entropy acquisition pipeline employing three MPU-9250/MPU-6500-class inertial sensors and von Neumann bias reduction to condition raw sensor-derived bitstreams [20].
- Dependency-aware characterization using official NIST SP 800-90B non-IID sequential estimators, together with an official NIST SP 800-22 STS 2.1.2 single-sequence assessment of conditioned output [21].
- A lightweight proof-of-concept implementation demonstrating practical deployment on a resource-constrained embedded platform.

The remainder of this paper is organized as follows. Section 2 describes the materials and methods. Section 3 presents the proposed EntroMEMS architecture. Section 4 describes the experimental methodology. Section 5 presents the experimental results and discussion. Finally, Section 6 concludes the paper.

## 2. Materials and Methods

### 2.1. Design Methodology

The development of the proposed EntroMEMS framework followed a structured methodology aimed at investigating the feasibility of combining quantum-generated initialization entropy with locally harvested physical entropy in a lightweight embedded security architecture. The study was designed to address the entropy limitations of resource-constrained edge devices by integrating two complementary entropy sources operating at different stages of the device lifecycle.

The proposed methodology consists of four phases. The first generates externally derived initialization material using IBM Quantum infrastructure. The second acquires candidate physical entropy from multiple MEMS gyroscopes. The third conditions and characterizes the resulting streams. The fourth quantifies statistical behavior, stability, and implementation cost. These phases demonstrate data flow and feasibility; they do not establish a protected production root of trust.

This layered methodology intentionally separates system design, experimental implementation, and performance evaluation. The architectural principles of the proposed framework are presented in Section 3, while the detailed experimental implementation and validation procedures are described in Section 4. The resulting statistical properties and security implications are subsequently analyzed in Section 5.

### 2.2. Hardware and Software Resources

The proposed EntroMEMS framework was implemented using a combination of commercially available embedded hardware, open-source software tools, and cloud-based quantum computing services. The embedded platform was responsible for local entropy acquisition and real-time processing, whereas the host computer executed the quantum communication, entropy conditioning, and statistical evaluation software. The principal hardware and software resources employed throughout the study are summarized in Table 1.

### 2.3. Quantum Computing Infrastructure

The quantum component of the proposed EntroMEMS framework was implemented using the IBM Quantum Platform, which provides cloud access to superconducting quantum processors through the Qiskit Runtime environment. This infrastructure enables the execution of quantum circuits on real quantum hardware without requiring dedicated local quantum resources.

For the experiments presented in this study, the ibm_fez quantum processor was employed to generate the initialization entropy used during the provisioning stage of the embedded device. Quantum circuit development and execution were performed using the Qiskit SDK, while Qiskit Runtime provided secure job submission and result retrieval. During software development and verification, the Qiskit Aer simulator was additionally used to validate circuit functionality before execution on the physical quantum processor.

The quantum platform was used during the laboratory initialization experiment. The current implementation does not demonstrate secure transport or protected storage of the resulting value. Production deployment would require authenticated provisioning, unique device identities, protected device-specific storage, disabled or authenticated debug access, key derivation, revocation, and reprovisioning.

## 3. Proposed EntroMEMS Architecture

### 3.1. System Overview

The EntroMEMS prototype is used to investigate how externally generated initialization material and locally acquired candidate physical entropy can coexist on a constrained edge platform. The demonstrated implementation is a laboratory proof of concept, not a complete trust architecture. In particular, protected provisioning, secure storage, authenticated communications, and a cryptographically approved DRBG were not implemented.

The complete architecture consists of five functional layers:

1. Quantum-derived initialization layer—A 1024-bit value is generated using repeated measurements of a single-qubit Hadamard circuit. In the laboratory prototype, it is embedded in firmware for reproducibility; a deployable design would require authenticated, unique per-device provisioning and protected storage.

2. Experimental generator layer—The first 128 bits of the stored value initialize Xorshift128+, which is retained only as a non-cryptographic experimental baseline and is not used to support claims of cryptographic security.

3. Local entropy acquisition layer—Three MPU-9250 inertial measurement units connected through a TCA9548A I^2^C multiplexer continuously provide raw sensor measurements. Low-order variations extracted from the gyroscope output are treated as candidate entropy samples and collected into a raw bitstream.

4. Entropy characterization layer—Raw sensor samples are analyzed separately by sensor and position. Conditioned output is evaluated with an official NIST SP 800-22 STS 2.1.2 single-sequence assessment covering all fifteen categories, while raw sequential samples are assessed with the official SP 800-90B non-IID estimators.

5. Telemetry demonstration layer—XOR masking demonstrates data-path integration only. It is not encryption, authenticated protection, or a substitute for a standard secure-communication protocol.

The prototype separates externally derived initialization material from local sensor acquisition. A production design would preserve this separation by feeding health-tested and conservatively credited local entropy through the reseed interface of a vetted DRBG rather than directly XORing raw sensor bits into output or overwriting prior state. Standardized DRBG mechanisms are specified in NIST SP 800-90A Rev. 1 [22].

Threat model and scope. The prototype evaluation considers accidental entropy degradation, predictable startup state, firmware extraction or cloning of an embedded constant, sensor or bus failure, and benign changes in orientation, mechanical excitation, and external heating. It does not experimentally evaluate invasive extraction, power or electromagnetic side channels, fault injection, malicious firmware, acoustic injection, supply manipulation, or a compromised cloud/provisioning endpoint. No resistance to these adversaries is claimed.

**Figure 1:**
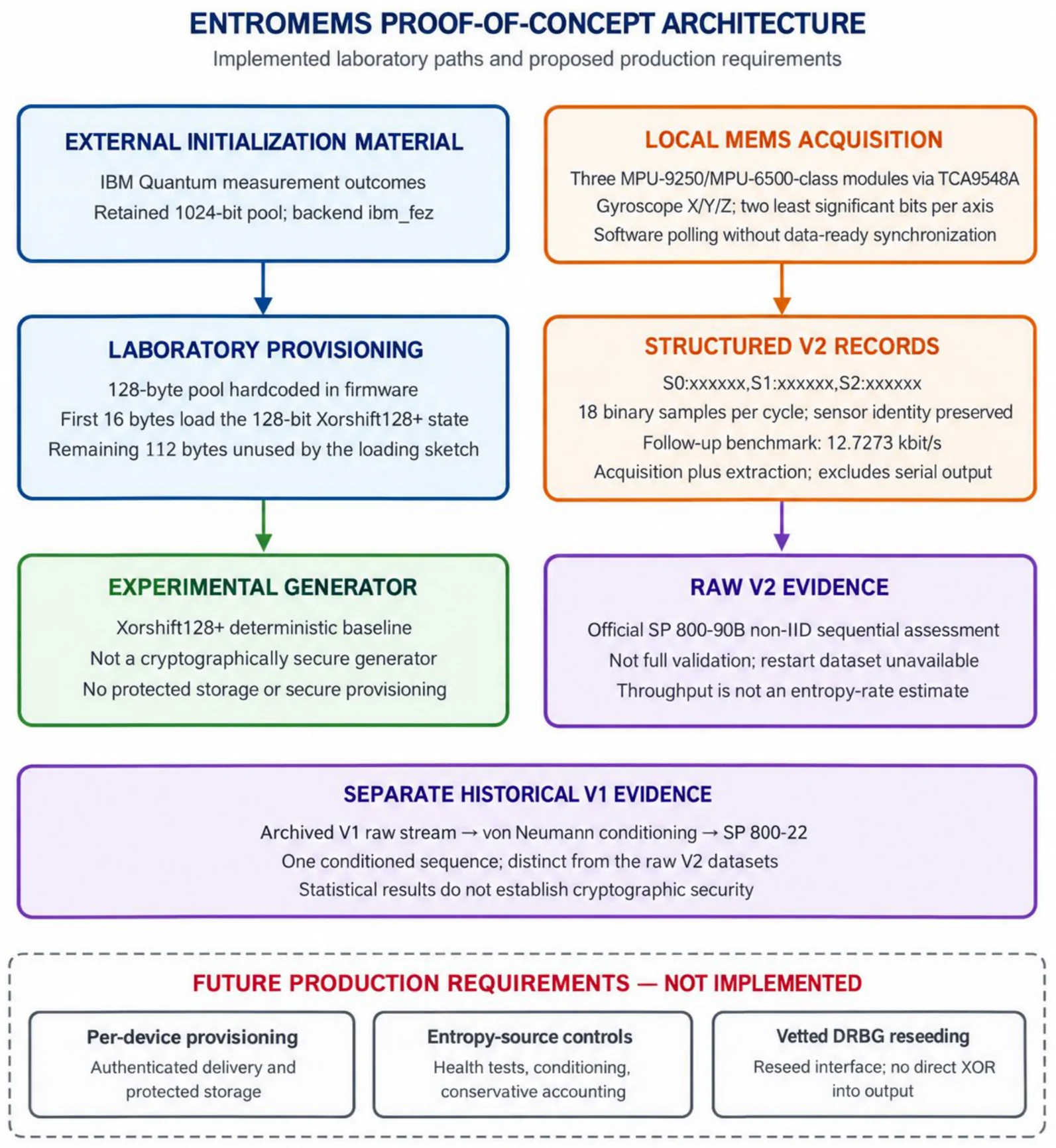
EntroMEMS system architecture: integration of quantum-derived seed provisioning and MEMS-based local entropy conditioning on a resource-constrained edge device.

The complete experimental workflow of the proposed EntroMEMS framework is illustrated in Figure 1.

### 3.2. Quantum Seed Provisioning

#### 3.2.1. Quantum Seed Generation

The EntroMEMS prototype uses quantum-derived material obtained from a cloud-accessible IBM Quantum processor to initialize its experimental generator. Unlike deterministic software initialization, quantum measurements originate from fundamentally probabilistic physical processes and therefore provide a suitable source of nondeterministic seed material for embedded security applications.

A single-qubit quantum circuit was employed to generate the initialization seed. Each circuit execution initializes the qubit in the computational basis state |0⟩, after which a Hadamard gate is applied to create an equal superposition prior to measurement:

(1) $$H|0\rangle =\frac{\left|0\rangle +\right|1\rangle }{\sqrt{2}}$$

The Hadamard transformation places the qubit into an equal superposition of both computational basis states. Subsequent measurement collapses the quantum state, producing a classical binary outcome with an ideal probability of

*P*(0) = *P*(1) = 0.5 (2)

Repeated measurements were performed on an operational IBM Quantum backend using the Qiskit Runtime environment [11,14]. The resulting binary measurement outcomes were concatenated to produce a 1024-bit initialization seed for the experimental edge generator; this does not establish a protected trust anchor.

#### 3.2.2. IBM Quantum Backend and Seed Provisioning

The 1024-bit quantum-derived value is serialized as a 128-byte array and embedded in the ESP32 firmware. At initialization, the retained loading sketch copies only the first 16 bytes into the 128-bit state of Xorshift128+; the remaining 112 bytes are not used by that sketch.

The laboratory provisioning stage supplies externally generated initialization material to the embedded device. In this prototype, the seed is embedded in firmware for reproducibility and does not provide secure device-specific initialization. Once provisioning is complete, the ESP32 operates autonomously without requiring continuous connectivity to the IBM Quantum infrastructure.

The architecture also remains compatible with future seed-refresh mechanisms based on either newly provisioned quantum-derived material or locally conditioned MEMS entropy. The detailed experimental implementation of the provisioning procedure is described in Section 4.2.

**Figure 2:**
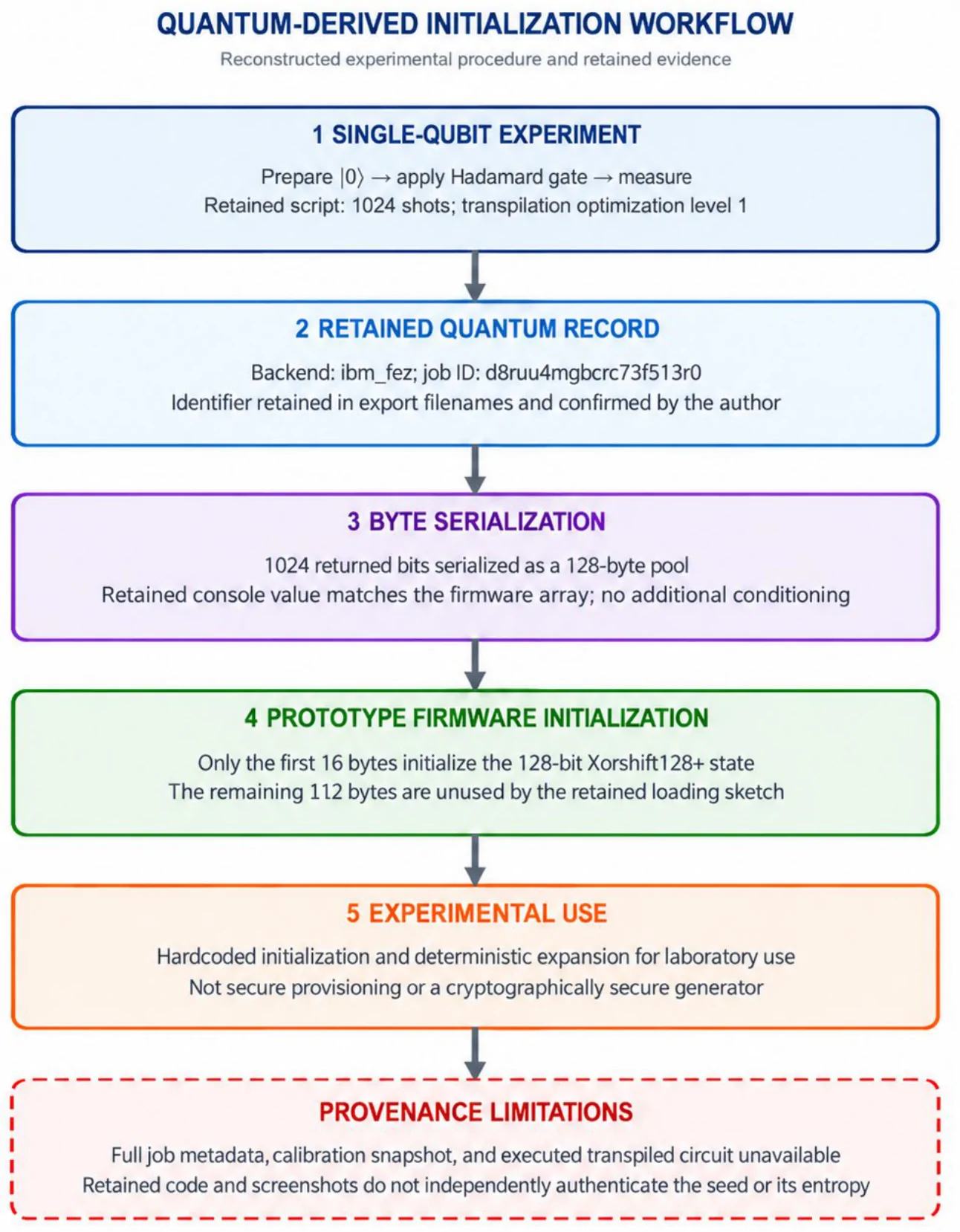
Quantum seed provisioning workflow from IBM Quantum cloud infrastructure to ESP32 edge device initialization. The 1024-bit quantum-derived seed is generated through repeated quantum measurements, serialized, provisioned to the embedded platform, and stored in firmware. Only the first 128 bits initialize the Xorshift128+ prototype state.

Figure 2 summarizes the transition from quantum-generated seed material to initialization of the embedded platform.

### 3.3. Local MEMS Entropy Acquisition and Conditioning

In addition to the externally provisioned quantum seed, the proposed EntroMEMS architecture incorporates a local entropy acquisition subsystem based on multiple MEMS inertial sensors. The objective of this subsystem is not to replace the quantum-derived initialization seed, but rather to investigate whether naturally occurring stochastic variations observed in low-cost MEMS devices can serve as an auxiliary entropy source for future reseeding and embedded security applications.

**Figure 3:**
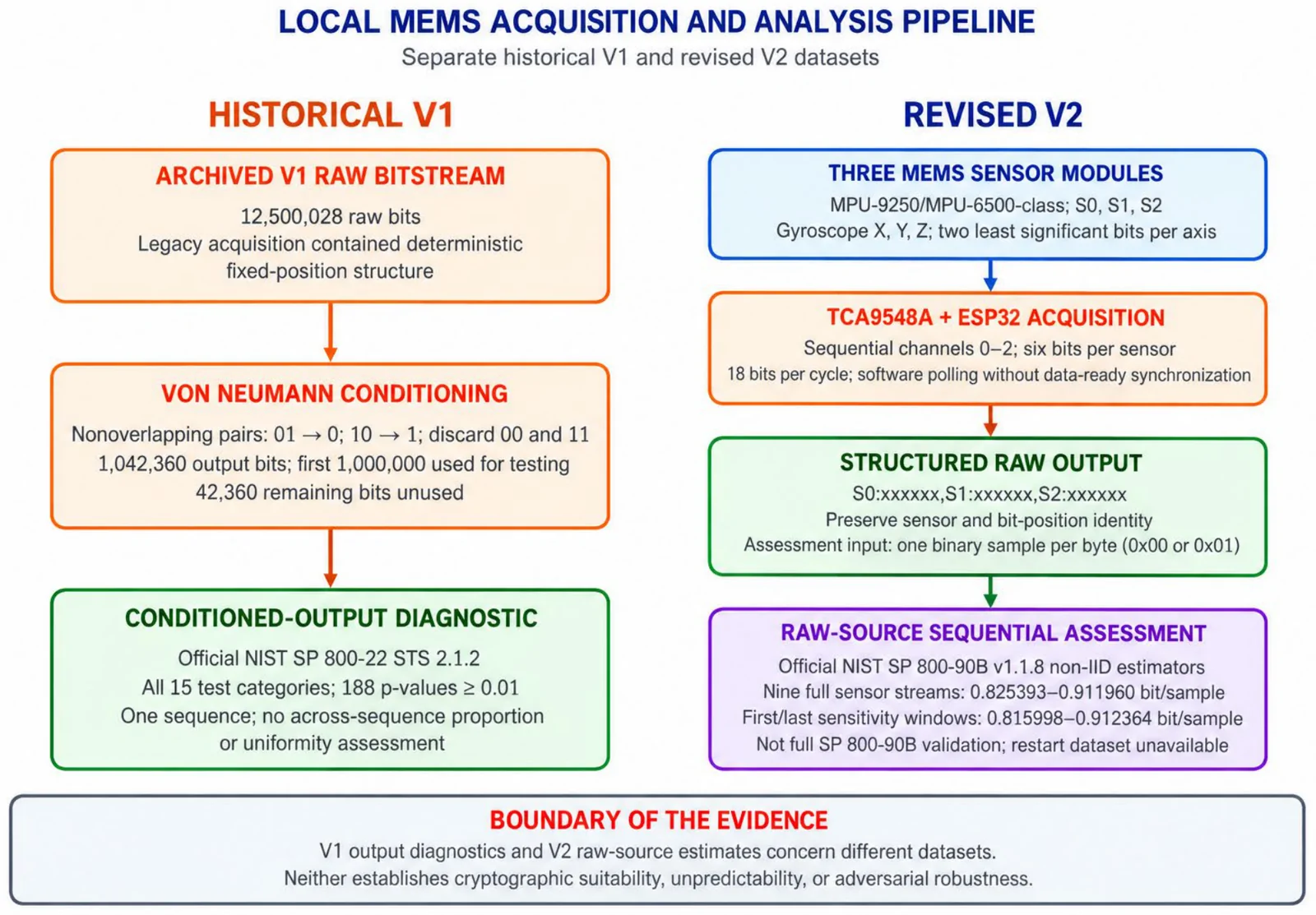
Local MEMS acquisition and conditioning pipeline.

Figure 3 distinguishes two experimental paths based on separate datasets. The historical V1 raw bitstream undergoes von Neumann conditioning, followed by an SP 800-22 single-sequence assessment of the conditioned output. The revised V2 acquisition preserves sensor and bit-position identity, and its unconditioned samples are evaluated separately using SP 800-90B non-IID sequential estimators. The V1 conditioned-output results are not used to establish the entropy of the V2 raw samples.

The experimental platform employs three MPU-9250/MPU-6500-class inertial sensor modules connected to an ESP32 microcontroller through a TCA9548A I^2^C multiplexer. The multiplexer enables sequential access to multiple sensors sharing the same I^2^C address while maintaining a compact hardware configuration suitable for resource-constrained edge devices. Geometrically redundant MEMS configurations have been investigated to reduce measurement errors [23]; here, the three sensor streams are retained separately for entropy characterization rather than combined to improve inertial measurement accuracy.

During each acquisition cycle, raw gyroscope measurements are obtained from all three sensors. Low-order bits extracted from the digital sensor output are collected and concatenated into a continuous binary stream. Because these measurements contain deterministic components together with sensor noise, quantization effects, and other stochastic variations, the resulting raw sequence is considered a candidate entropy source rather than directly usable cryptographic material.

The official NIST SP 800-22 STS 2.1.2 execution produced 188 individual *p*-values across all fifteen categories for the first contiguous 1,000,000 conditioned bits; all were at least 0.01. This was a single-sequence assessment, so across-sequence proportion and uniformity criteria were not assessed. For raw V2 samples, official NIST SP 800-90B v1.1.8 non-IID sequential H_original estimates ranged from 0.825393 to 0.911960 bit/sample across nine full per-sensor runs; estimates from the sensitivity windows ranged from 0.815998 to 0.912364 bit/sample. The lowest observed estimate, 0.815998 bit/sample, is not presented as a validated population lower bound.

The conditioned output was assessed with Shannon entropy and an official NIST SP 800-22 STS 2.1.2 single-sequence run covering all fifteen categories. This conditioned-output assessment is kept separate from the dependency-aware assessment of unconditioned V2 samples.

The acquired MEMS bitstream is treated as a candidate physical entropy input to the proposed framework. Since the statistical properties of the raw sequence are influenced by sensor noise and measurement bias, an entropy conditioning stage is subsequently applied before the bitstream is subjected to statistical evaluation. The conditioning procedure is described in Section 4.4, while the corresponding experimental results are presented and quantitatively analyzed in Section 5.

### 3.4. Edge Security Layer

The final layer of the proposed EntroMEMS architecture demonstrates data-path integration on the embedded edge device. The ESP32 initializes its experimental deterministic generator using the first 128 bits of the externally generated 1024-bit pool embedded in firmware. The resulting output is used for the XOR telemetry-masking demonstration; this does not establish a protected trust state or authenticated communication.

To demonstrate the practical applicability of the proposed architecture, the initialized random stream is employed as an experimental masking mechanism for MEMS telemetry. The masking operation is implemented using a lightweight XOR-based transformation, allowing the transmitted sensor values to be combined with the locally generated random stream before communication. The objective of this implementation is to demonstrate the feasibility of integrating externally provisioned quantum-derived initialization with real-time embedded telemetry processing on a resource-constrained platform.

The current prototype employs the Xorshift128+ algorithm as a lightweight deterministic random bit generator owing to its small memory footprint and computational efficiency on the ESP32 platform. It should be emphasized that Xorshift128+ is used exclusively as an experimental baseline implementation and is not presented as a cryptographically secure deterministic random bit generator. The proposed architecture is independent of the specific generator implementation and can be extended in future work by replacing the current baseline with standardized cryptographically secure deterministic random bit generators.

Following initialization, the embedded platform operates autonomously without requiring continuous connectivity to the IBM Quantum infrastructure. This design minimizes communication overhead while remaining compatible with future extensions, including adaptive reseeding and entropy health monitoring.

## 4. Experimental Methodology

This section describes the experimental setup, hardware platform, software environment, and statistical evaluation procedures used to validate the proposed EntroMEMS architecture. The experiments were designed to evaluate both the cloud-to-edge quantum seed provisioning workflow and the local MEMS-derived entropy acquisition pipeline implemented on a resource-constrained embedded platform.

### 4.1. Hardware Platform

The hardware and software resources used throughout this study are summarized in Table 1 (Section 2.2). This section focuses on their integration into the experimental platform and the procedures used for validation.

The experimental prototype was implemented on an ESP32 development board serving as the central embedded processing unit. The ESP32 was selected because of its widespread adoption in Industrial Internet of Things (IIoT) applications, low power consumption, integrated wireless connectivity, and sufficient computational resources for real-time sensor processing.

The local entropy acquisition subsystem consisted of three MPU-9250/MPU-6500-class inertial sensor modules connected to the ESP32 through a TCA9548A I^2^C multiplexer. Since all MPU-9250/MPU-6500-class sensors share the same default I^2^C address, the multiplexer enabled sequential communication with each device while maintaining a simple and compact hardware architecture. During each acquisition cycle, raw gyroscope measurements were collected from all three sensors and processed by the ESP32 to extract low-order candidate entropy bits.

The embedded firmware, implemented with the Arduino framework, performs TCA9548A channel selection, MPU gyroscope register reads, two-LSB extraction, structured record formation, and the timing/resource benchmarks on the ESP32. The host computer captures the serial stream and performs file parsing, von Neumann conditioning, calculations of Shannon entropy and empirical marginal min-entropy, NIST SP 800-22 execution, NIST SP 800-90B non-IID assessment, and report generation. Statistical evaluation is therefore offline and is not presented as an on-device implementation.

Quantum-derived initialization material was generated using the IBM Quantum cloud platform through the Qiskit Runtime environment. The resulting 1024-bit value was exported as a binary byte array and embedded in the ESP32 prototype firmware. The local MEMS acquisition was performed independently of this value.

**Figure 4:**
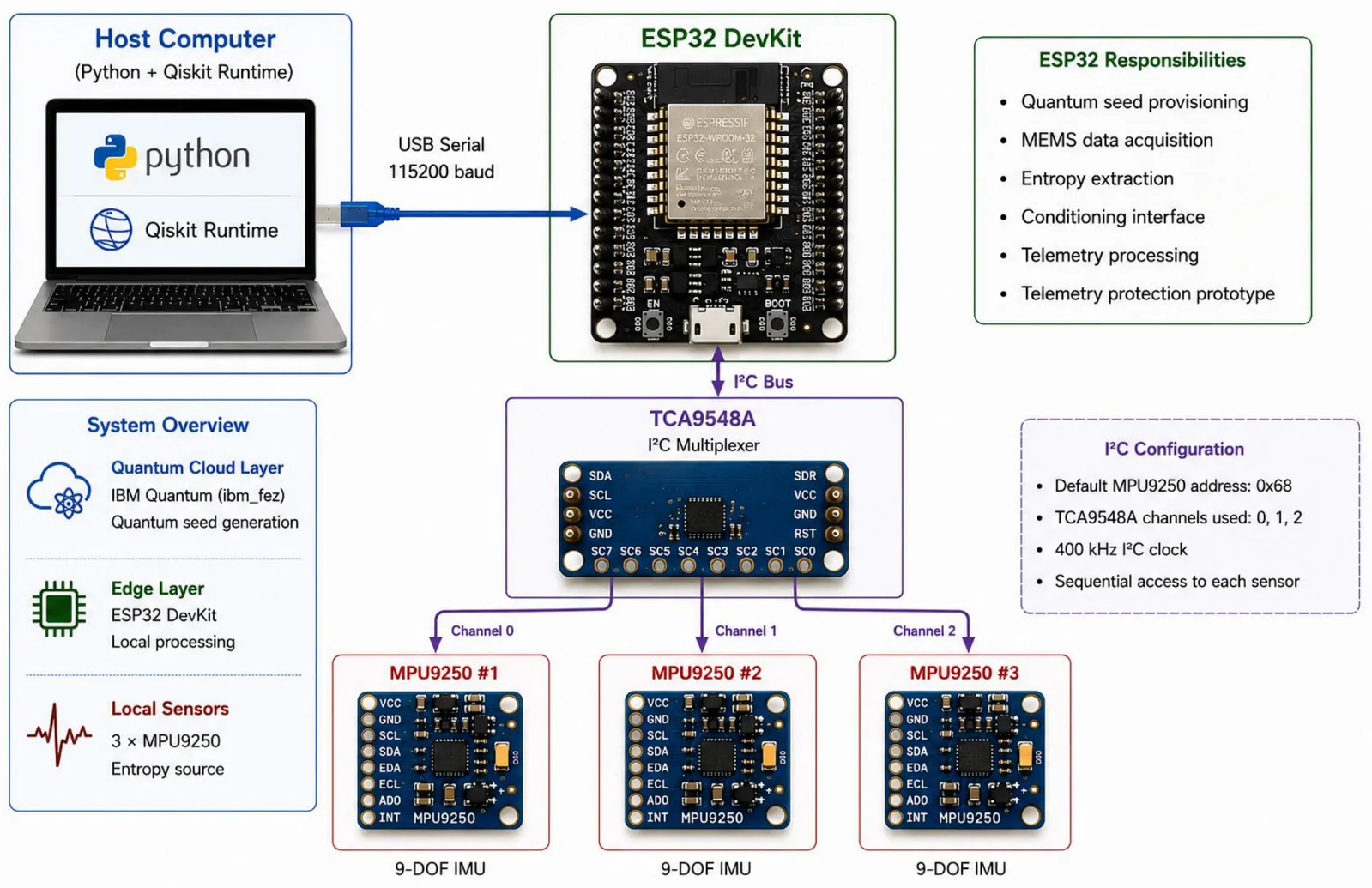
Experimental hardware configuration of the proposed EntroMEMS prototype. The host computer executes the quantum provisioning and data-processing software, while the ESP32 interfaces with three MPU-9250/MPU-6500-class sensors through a TCA9548A I^2^C multiplexer for local entropy acquisition and embedded processing.

**Figure 5:**
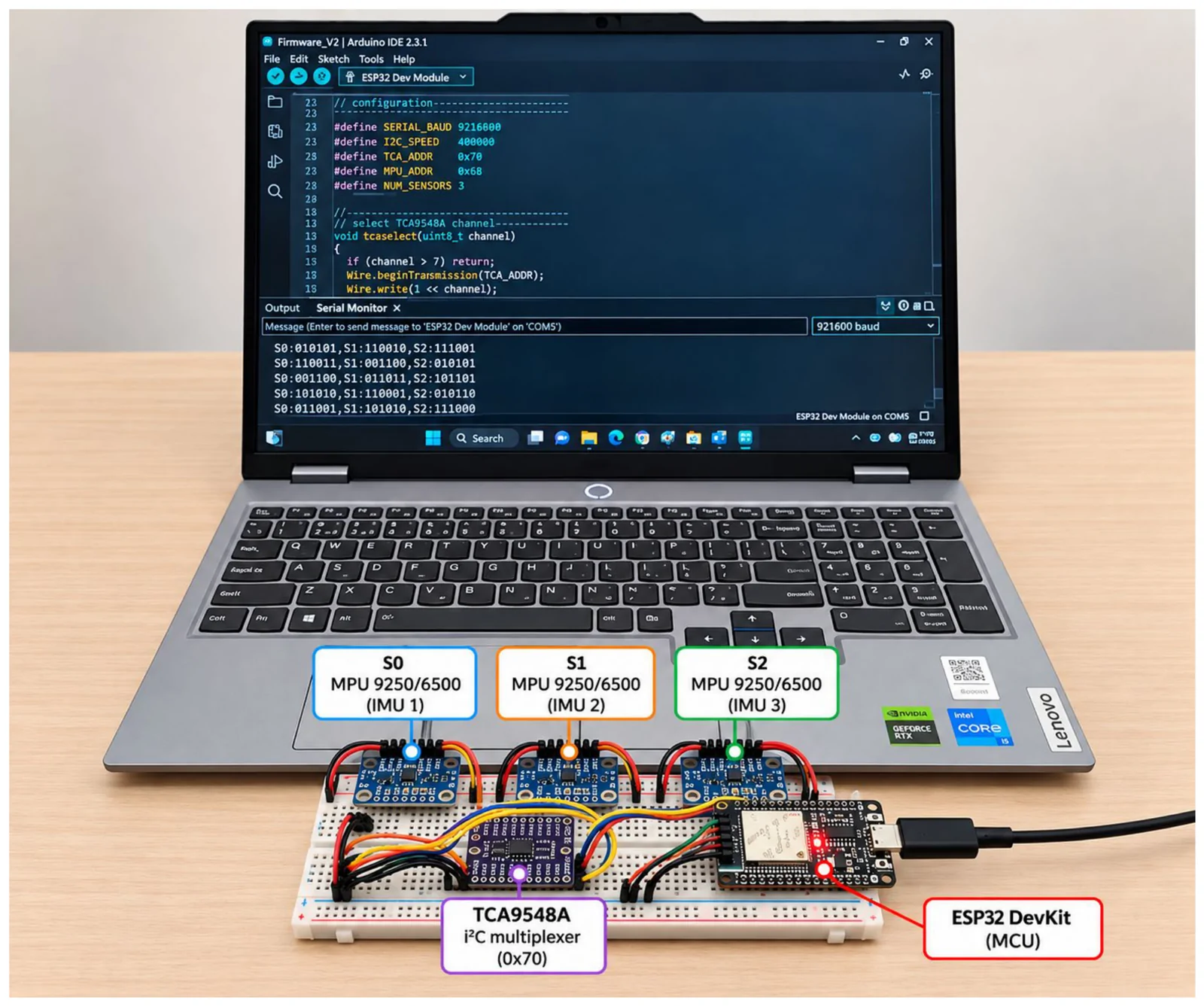
Background-cleaned and annotated photograph of the experimental EntroMEMS V2 prototype used in this study. The setup consists of an ESP32 development board connected to three MPU-9250/6500 inertial measurement units through a TCA9548A I^2^C multiplexer. The host computer runs the acquisition interface and records the structured raw gyroscope-LSB streams from sensors S0, S1, and S2.

The physical arrangement of the experimental platform and the interconnections among the host computer, ESP32 edge device, I2C multiplexer, and MEMS sensors are illustrated in Figure 4 and Figure 5.

### 4.2. Quantum Seed Generation and Provisioning

The externally generated initialization material was obtained through the IBM Quantum cloud platform using the Qiskit Runtime environment and the ibm_fez backend. The experiment demonstrates a provisioning data path; it does not establish a protected trust anchor.

A single-qubit quantum circuit was employed to generate the initialization seed. The qubit was initialized in the computational basis state |0⟩, transformed into an equal superposition by applying a Hadamard gate, and subsequently measured in the computational basis. The resulting binary measurement outcomes were concatenated to produce a 1024-bit initialization seed.

The retained Python 3.13.5 script requests 1024 shots of a single-qubit Hadamard-and-measurement circuit through Qiskit Runtime SamplerV2. It selects an operational non-simulator backend using least_busy and transpiles the circuit at optimization level 1. The archived run is identified as job d8ruu4mgbcrc73f513r0, based on the retained histogram export filenames and author confirmation. A retained dashboard screenshot reports a completed execution on ibm_fez, created on 21 June 2026 at 5:02 PM (display timezone not recorded), and a Usage value of 2 s. This field is not an end-to-end provisioning-latency measurement. The script concatenates the returned one-bit shot strings and converts them to hexadecimal without additional conditioning.

The original account is no longer accessible. The complete machine-readable job metadata, backend calibration snapshot, physical-qubit mapping, executed transpiled circuit, and exact historical Qiskit package versions were not recovered. The histogram does not retain shot order. The retained code and screenshots support procedural traceability but do not independently authenticate the particular seed or validate its entropy; the value is therefore described as quantum-derived initialization material rather than certified QRNG output.

The generated value was exported as a 128-byte hexadecimal array and embedded in the ESP32 firmware as QUANTUM_SEED_POOL [128]. This hardcoding enabled repeatable laboratory experiments but offers no protection against firmware extraction, cloning, invasive access, or side-channel attacks. It must not be interpreted as secure key storage or a protected root of trust. The recovered console value matches this array exactly. The loading sketch copies only its first 16 bytes into the 128-bit Xorshift128+ state.

A deployable design would generate or derive unique material for each device, authenticate both endpoints during provisioning, protect storage using a secure element or appropriately configured encrypted flash, restrict debug interfaces, and support revocation and reprovisioning. Side-channel resistance would require separate implementation-specific evaluation and is not demonstrated here.

**Figure 6:**
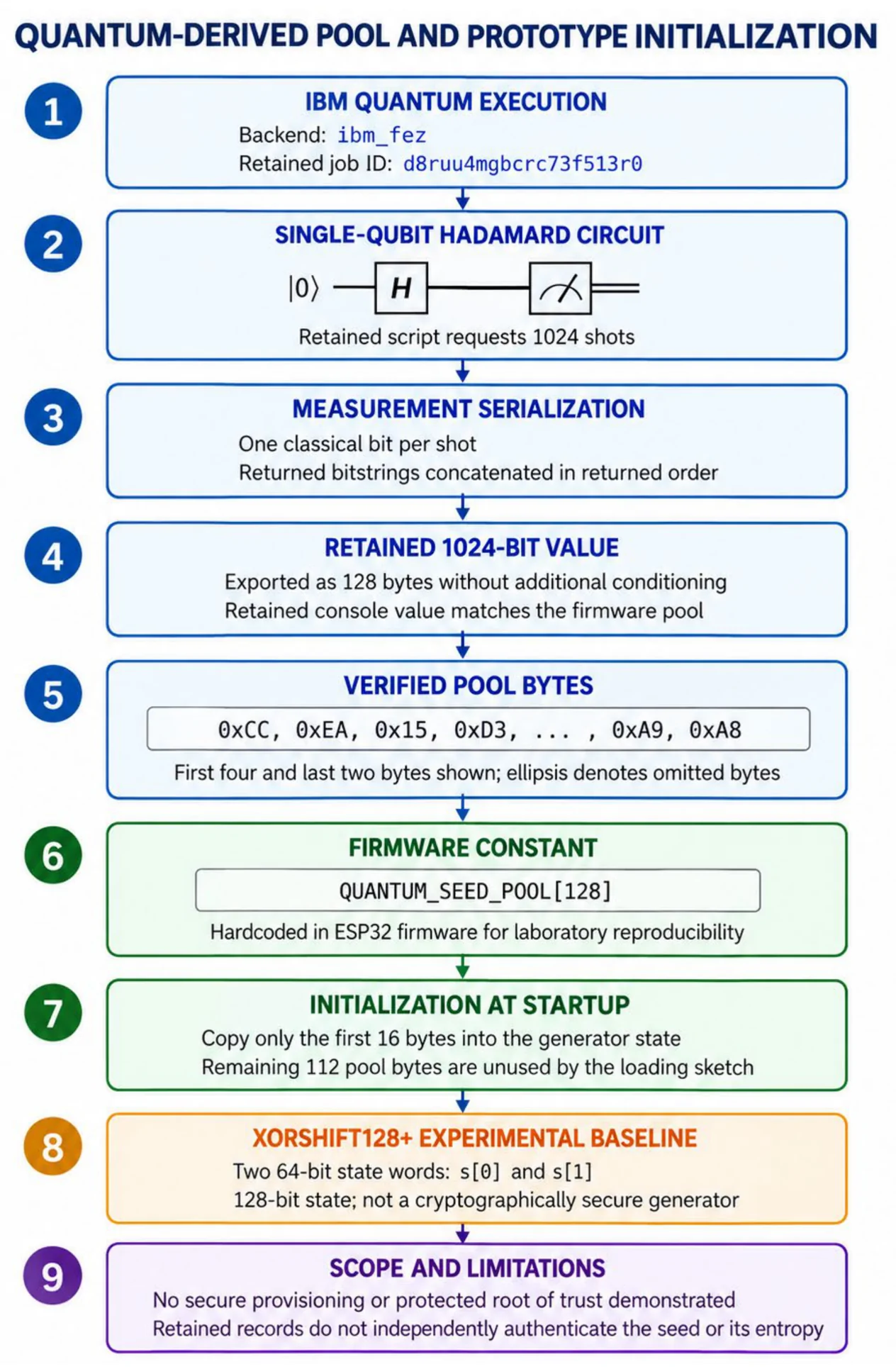
Quantum seed generation and provisioning workflow implemented in the proposed EntroMEMS architecture. A 1024-bit seed is generated from repeated measurements of a single-qubit Hadamard circuit executed on the IBM Quantum ibm_fez backend, exported as a 128-byte hexadecimal array, embedded into the ESP32 firmware as QUANTUM_SEED_POOL [128], and retained as a pool. Only the first 16 bytes initialize the 128-bit Xorshift128+ state.

The complete quantum seed generation and provisioning workflow is illustrated in Figure 6.

Figure 6 summarizes the laboratory workflow from quantum measurement serialization to initialization of the experimental Xorshift128+ generator. Only the first 16 bytes of the retained 128-byte pool initialize its state. Hardcoded initialization does not establish secure provisioning, protected storage, or a quantum root of trust.

### 4.3. MEMS Entropy Acquisition

Local entropy was acquired using three MPU-9250/MPU-6500-class inertial sensor modules connected to the ESP32 through a TCA9548A I^2^C multiplexer. Since all sensors share the same default I^2^C address (0 × 68), the multiplexer enabled sequential communication with each device without address conflicts.

In V2, the ESP32 selects TCA9548A channels 0–2 sequentially and reads six bytes beginning at gyroscope register 0 × 43 from each device, corresponding to Gyro X, Y, and Z. The two least significant bits are retained from each signed 16-bit axis word without applying abs(), yielding six binary samples per sensor and 18 per complete cycle. Serial records preserve sensor identity as S0:xxxxxx, S1:xxxxxx, S2:xxxxxx.

The V2 acquisition firmware used a 400 kHz I2C bus. Structured serial records were captured by the host without packing the binary samples. For SP 800-90B assessment, each extracted bit was stored as one full byte, 0 × 00 or 0 × 01; ASCII characters and packed-bit files were not used.

The archived V2 firmware writes 0 × 00 to PWR_MGMT_1 but does not explicitly set or read back GYRO_CONFIG, CONFIG, or SMPLRT_DIV. It polls the three sensors sequentially without waiting for data-ready or imposing a fixed sampling period. Historical configuration logs and per-read timestamps were not retained; freshness at every historical read cannot therefore be established retrospectively. The sequential entropy estimates characterize the recorded software-polled streams.

A follow-up audit on 7 October 2026 reported WHO_AM_I = 0 × 70, GYRO_CONFIG = 0 × 00, CONFIG = 0 × 00, and SMPLRT_DIV = 0 × 00 on all three modules after the V2 power-management write. The identification readback is consistent with MPU-6500-class devices. Interpreted using the MPU-6500 register map (RM-MPU-6500A-00, Revision 2.1; source documented in the Supplementary Materials), these settings select ±250 degrees/s, DLPF_CFG = 0 with a nominal 250 Hz gyroscope bandwidth, and a nominal internal rate of 8 kHz. These are configuration-based values, not measured conversion frequencies or proof of the September settings.

A separate instrumented follow-up recorded three batches of 1000 three-sensor cycles. Baseline batch means were 1416.669, 1416.662, and 1416.659 microseconds per cycle. With the raw-data-ready indication enabled and INT_STATUS read before each gyroscope transaction, means were 2076.924, 2076.900, and 2076.937 microseconds. The data-ready bit was set in all 9000 status observations, supporting register updates between status observations in this diagnostic mode. The flag neither counts conversions nor establishes sample independence or historical freshness. Extra status transactions change the workload; the original interrupt settings were restored and verified afterward.

The revision uses three independent five-minute baseline acquisitions following separate startup cycles. Each sensor stream contains approximately 1.20–1.23 million sequential binary samples. Additional runs evaluated static yaw orientations of 90 and 180 degrees, a 90-degree tilt, a staged mechanical perturbation sequence, and a staged sequence involving moderate external heating.

**Figure 7:**
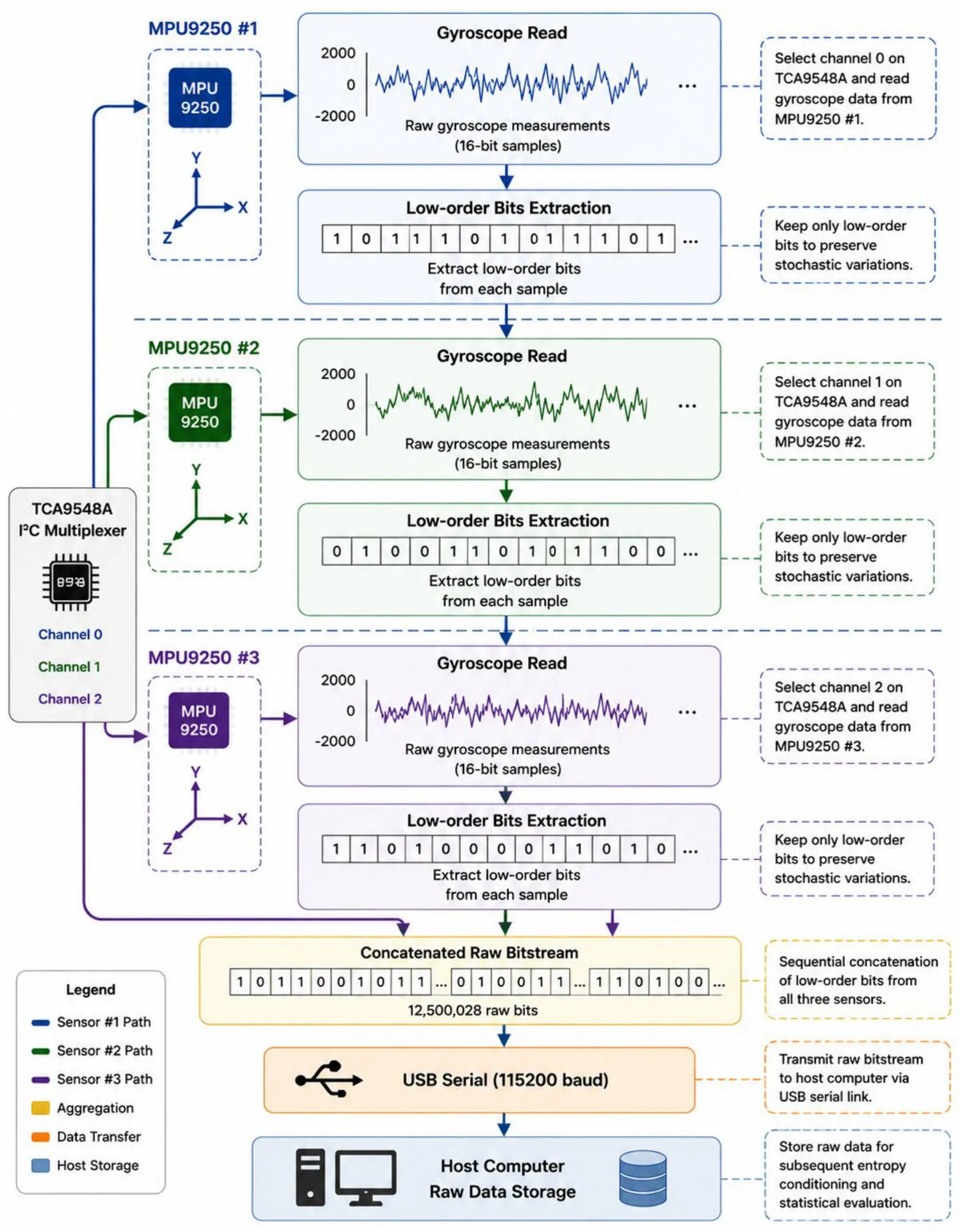
Historical V1 MEMS acquisition workflow. Raw gyroscope measurements are sequentially collected from three MPU-9250/MPU-6500-class sensors through the TCA9548A I^2^C multiplexer. Low-order bits extracted from each sensor are concatenated into a continuous raw bitstream comprising 12,500,028 bits and transmitted to the host computer via a USB serial interface for subsequent entropy conditioning and statistical evaluation. The plotted traces and bit patterns are schematic, not measured values.

Figure 7 summarizes the historical V1 acquisition workflow; the revised V2 acquisition procedure is described above.

### 4.4. Entropy Conditioning

Two distinct datasets are retained. The historical V1 dataset is used only to document the original von Neumann conditioning and SP 800-22 workflow. V2 corrects the acquisition model by reading all three gyroscope axes explicitly and preserving sensor identity. Raw V2 samples are assessed before conditioning with dependency-aware non-IID estimators.

To reduce the bias in the historical V1 bitstream, the classical von Neumann extraction algorithm was applied to the complete raw bitstream. The algorithm processes the input as consecutive pairs of bits according to the following rules: pairs 01 and 10 are mapped to output bits 0 and 1, respectively, while pairs 00 and 11 are discarded. By eliminating identical bit pairs, the procedure reduces first-order bias through deterministic processing of the input bit pairs.

Conditioning and statistical evaluation were performed offline on the host computer. Von Neumann extraction can reduce first-order bias under appropriate assumptions, but it does not by itself prove independence or quantify source min-entropy. Accordingly, the revised study reports conditioned-sequence tests separately from raw-source entropy estimates.

The conditioned bitstream served as input to Shannon entropy calculation and the official NIST SP 800-22 STS 2.1.2 single-sequence assessment described below. Raw V2 samples were analyzed separately with non-IID entropy estimators.

The Supplementary Materials include the retained V1 raw file and original conditioning script, plus a portable reproduction check. Applying the original function to all 12,500,028 raw bits produces 1,042,360 conditioned bits; its first 1,000,000 bits match the retained NIST input byte for byte. The original script name and plot labels refer to quantum processing, but this input is the historical MEMS V1 stream.

**Figure 8:**
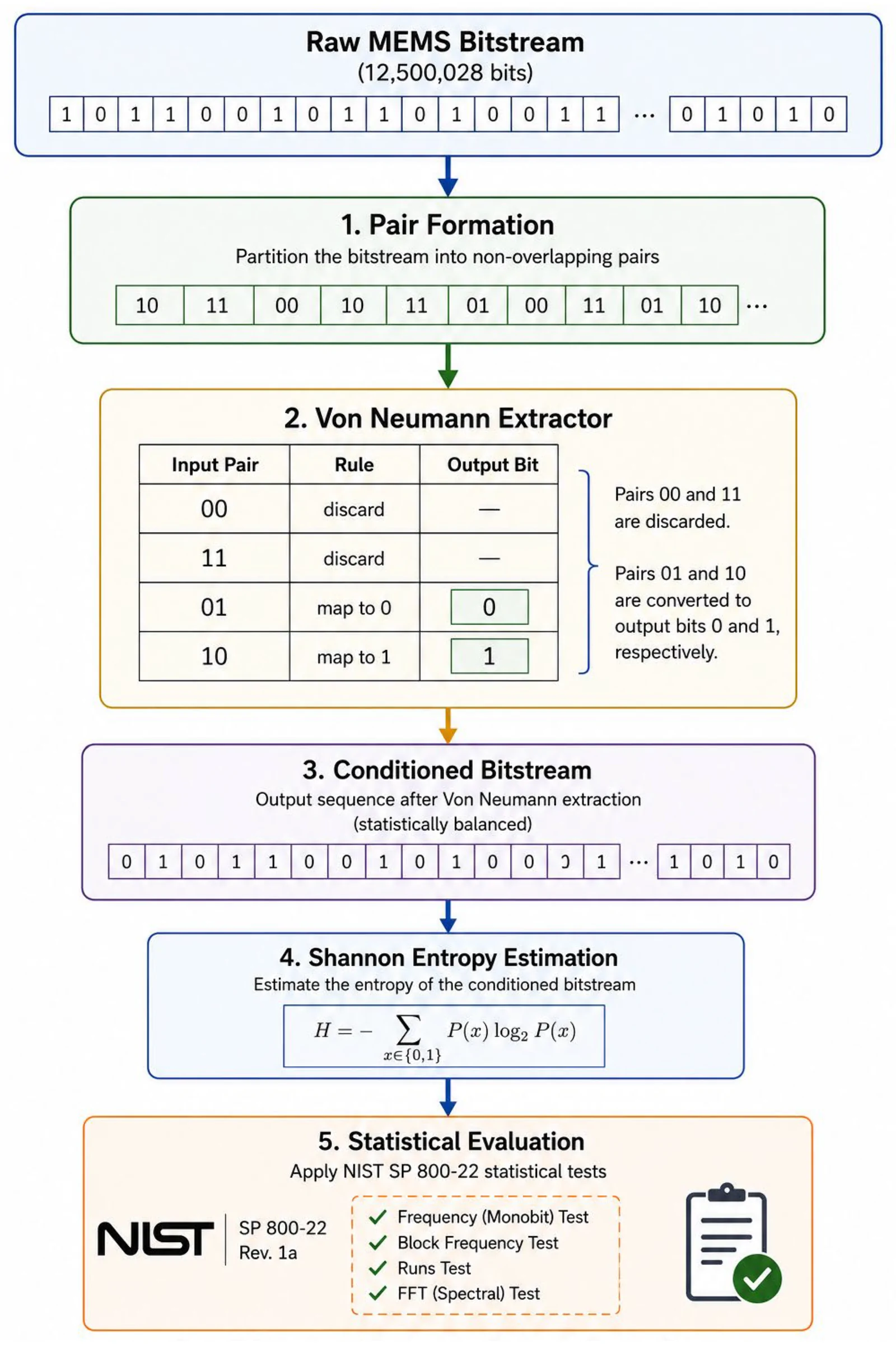
Entropy conditioning and statistical evaluation workflow. Von Neumann processing is applied to the historical V1 stream for conditioned-output diagnostics. Raw V2 streams are preserved separately for dependency-aware NIST SP 800-90B non-IID sequential assessment.

Figure 8 summarizes the entropy conditioning and statistical evaluation workflow, distinguishing the historical V1 conditioning and output assessment from the non-IID sequential assessment of raw V2 samples.

### 4.5. Statistical Evaluation

The evaluation separates three concepts: Shannon entropy and empirical marginal min-entropy describe symbol frequencies; NIST SP 800-22 tests detect selected statistical deviations in a finite conditioned sequence; and NIST SP 800-90B non-IID estimators provide dependency-aware sequential min-entropy estimates for raw samples. None of these alone constitutes full entropy-source validation. Statistical-test dependence, error, sensitivity, and uniformity have been examined in prior work [24,25,26].

The Shannon entropy of the conditioned bitstream was calculated to estimate the average information content per generated bit. For a binary source, the entropy is defined as

*H*(*X*) = −*sum*_{*x in* {0,1}} *P*(*x*) *log*_2 *P*(*x*). (3)

where P(0) and P(1) are the empirical probabilities of binary values 0 and 1, respectively [1,2]. This marginal quantity does not account for temporal or structural dependence.

The first contiguous 1,000,000 conditioned bits, selected before inspection of the test outcomes, were evaluated as a single ASCII sequence using the official NIST SP 800-22 STS 2.1.2 software with default parameters and alpha = 0.01. The assessment covered all fifteen test categories, including Universal, Linear Complexity, and Approximate Entropy. The complete console transcript, final report, and per-test results were retained. Individual *p*-values are interpreted only as evidence that the corresponding test instance did not reject the null hypothesis for this sequence. Across-sequence pass proportions and *p*-value uniformity are not reported because only one sequence was evaluated.

Raw V2 streams were evaluated with the official NIST SP 800-90B EntropyAssessment software v1.1.8 (repository commit 68ed165fd7a3eeef26b87a546ba23f338e82a3f3), using the non-IID track. Nine full per-sensor baseline streams and eighteen first/last one-million-sample sensitivity windows were assessed. The first and last windows overlap and are used only as sensitivity checks.

Table 2 distinguishes measured API/acquisition throughput from conceptual functional comparisons. The measured rates exclude secure reseeding and end-to-end communication. The table does not compare validated entropy rates or rank cryptographic security.

### 4.6. Follow-Up Timing Method

Performance was remeasured on 7 October 2026 with an ESP32 at 240 MHz, Arduino-ESP32 3.3.11, ESP-IDF v5.5.5, compiler 14.2.0, and I2C at 400 kHz. The new benchmark explicitly wakes the sensors and checks their registers and read errors. Each timed batch is bracketed by micros(), which wraps esp_timer_get_time() in this core and returns integer microseconds (1 microsecond timer granularity). Serial output is drained before timing, and no serial printing occurs within a timed batch. Acquisition-only and acquisition-plus-extraction tests each use 10,000 three-sensor cycles; extraction and esp_random() tests each use 1,000,000 iterations. Three sequential repetitions were performed within one boot.

For each run, elapsed microseconds are divided by the iteration count; reported means and sample standard deviations are calculated across the three resulting batch means (sample-variance denominator n − 1). Submicrosecond averages arise from batch division, not submicrosecond timer resolution. The extraction loop includes input updates, loop control, and volatile XOR accumulation; the RNG loop also includes accumulation overhead. The acquisition benchmark excludes ASCII record formation and serial transmission. The original benchmark source is retained, but the individual historical reports needed to verify 1411.043 ± 0.051 microseconds and 0.230 microseconds were not recovered. The follow-up results replace those earlier performance figures and are not measurements from the historical acquisition sessions.

## 5. Results and Discussion

This section reports the original conditioning experiment, the official SP 800-22 single-sequence assessment, the V2 non-IID sequential estimates, stability observations, and embedded-platform benchmarks. Statistical observations are not interpreted as proof of production cryptographic security.

### 5.1. Statistical Properties of the Conditioned Bitstream

The raw MEMS-derived dataset contained 12,500,028 bits acquired from the three MPU-9250/MPU-6500-class sensors. Before conditioning, the sequence exhibited a pronounced statistical bias, with approximately 83% zeros and 17% ones. The corresponding Shannon entropy was 0.6500918889 bits per symbol, indicating that the raw sequence was not sufficiently balanced for direct use as an entropy source.

The complete raw dataset was subsequently processed using the von Neumann extractor. The conditioning procedure produced more than one million output bits, from which a sequence of exactly 1,000,000 bits was selected for statistical evaluation. An additional 42,360 conditioned bits remained unused.

The evaluated conditioned sequence exhibited an almost perfectly balanced distribution, with

*P*(0) = 0.500111 (4)

and

*P*(1) = 0.499889. (5)

The Shannon entropy was calculated from these probabilities as

*H* = 0.9999999644 bits per symbol. (6)

This value is extremely close to the theoretical maximum of one bit per symbol for a balanced binary source. The results therefore demonstrate that the von Neumann conditioning stage substantially reduced the first-order bias present in the raw MEMS-derived bitstream.

**Figure 9:**
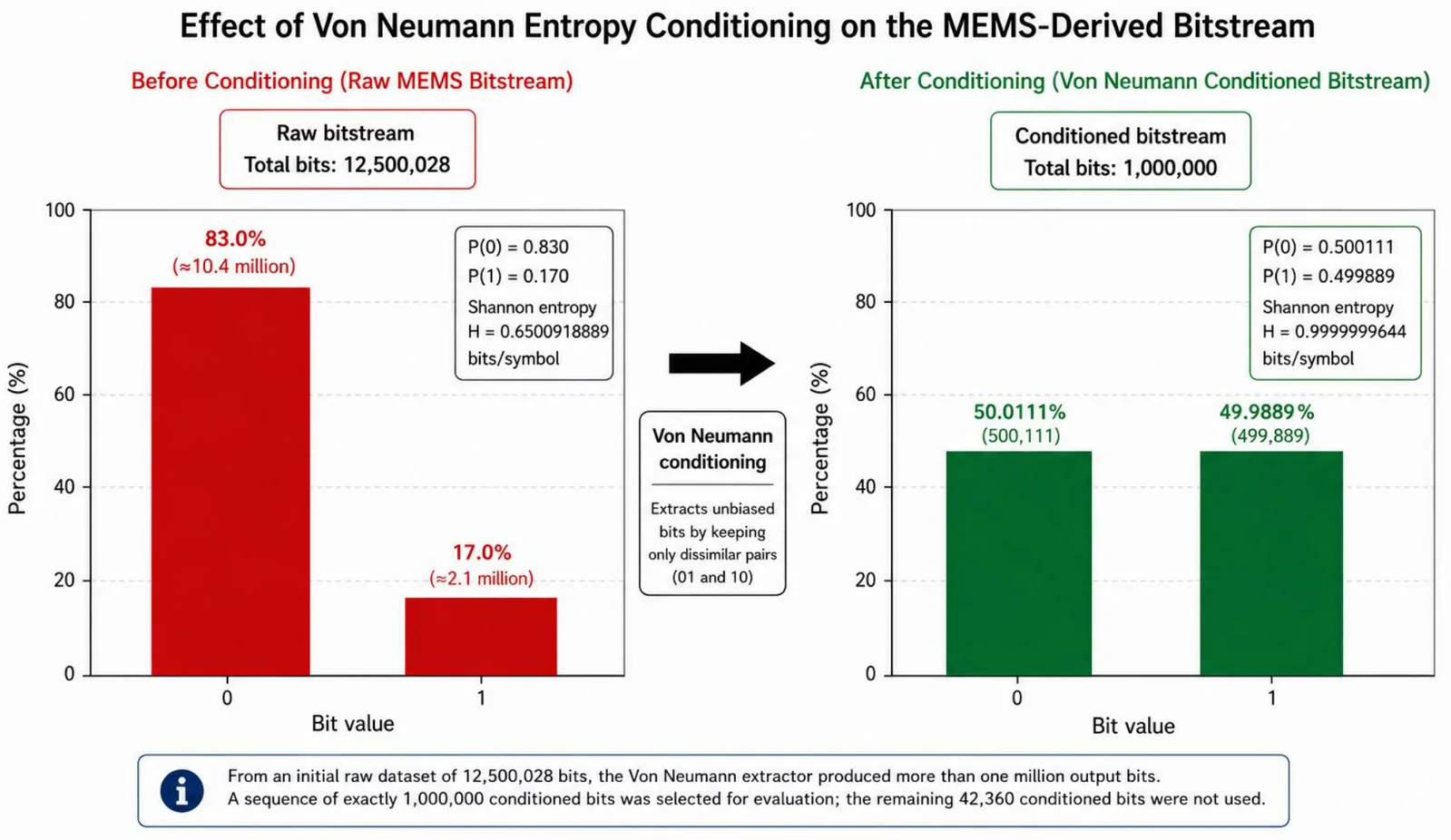
Effect of von Neumann conditioning on the historical V1 MEMS-derived bitstream. The raw dataset comprised 12,500,028 bits and exhibited approximately 83% zeros and 17% ones, with Shannon entropy 0.6500918889 bit/symbol. The first contiguous 1,000,000 conditioned bits had P(0) = 0.500111, P(1) = 0.499889, and a Shannon entropy of 0.9999999644 bit/symbol. These marginal statistics do not constitute a dependency-aware entropy estimate.

The statistical properties before and after conditioning are summarized in Table 3 and illustrated in Figure 9.

### 5.2. NIST SP 800-22 Statistical Evaluation

The first contiguous 1,000,000 conditioned bits were evaluated as a single ASCII sequence using the official NIST SP 800-22 STS 2.1.2 software with default parameters and alpha = 0.01. The assessment covered all fifteen test categories. The sequence length supports the configured Universal, Linear Complexity, and Approximate Entropy tests. The complete console transcript, final report, per-test statistics, and individual *p*-values were retained.

The complete battery comprised Frequency, Block Frequency, Runs, Longest Run, Binary Matrix Rank, Discrete Fourier Transform, Non-overlapping Template, Overlapping Template, Maurer’s Universal, Linear Complexity, Serial, Approximate Entropy, Cumulative Sums, Random Excursions, and Random Excursions Variant.

Table 4 summarizes the individual test results by category. The suite produced 188 *p*-values, all at least alpha = 0.01; categories containing multiple components are summarized by their *p*-value range and the number of passing test instances.

For the evaluated 1,000,000-bit conditioned sequence, all 188 individual *p*-values were at least alpha = 0.01, ranging from 0.010783 to 0.999360. Thus, no individual test instance rejected the null hypothesis at the configured threshold. Because only one sequence was evaluated, the NIST across-sequence pass-proportion and *p*-value-uniformity criteria are not meaningfully assessable. The result does not estimate source entropy or establish cryptographic suitability.

NIST SP 800-22 is therefore used as a conditioned-output diagnostic and is not substituted for entropy-source assessment.

### 5.3. SP 800-90B Non-IID Sequential Assessment

For the nine full per-sensor baseline streams, H_original ranged from 0.825393 to 0.911960 bit/sample. Across eighteen first/last one-million-sample sensitivity windows, estimates ranged from 0.815998 to 0.912364 bit/sample. The Compression estimator was the limiting estimator in all nine full assessments and in seventeen sensitivity windows; the Collision estimator was limiting in one sensitivity window. The empirical marginal min-entropy, −log2(max(P(0),P(1))), is reported separately and must not be confused with the SP 800-90B non-IID estimate.

The assessments were performed following NIST SP 800-90B [27], using the official NIST SP 800-90B EntropyAssessment software, version 1.1.8 (commit 68ed165fd7a3eeef26b87a546ba23f338e82a3f3). Inputs contained one binary symbol per byte (0 × 00 or 0 × 01). Exact commands, version information, and raw outputs were retained. Because no 1000 × 1000 restart dataset was collected, these results are sequential non-IID assessments and not full SP 800-90B validation.

### 5.4. Stability and Environmental Observations

Sensor and bit-position identities were preserved in three independent baseline acquisitions and additional yaw, tilt, mechanical-perturbation, and moderate-heating runs. Across the tested conditions, combined P(1) remained close to 0.5, Shannon entropy remained close to one bit per binary symbol, and lag-1 correlations remained close to zero. The most extreme position-level descriptive values were a marginal min-entropy of 0.991182 bit, absolute lag-1 correlation of 0.005330, pooled cross-position correlation of 0.003612, and lag-1-to-36 autocorrelation of 0.002756. No bit position was observed to remain constant.

These observations are limited to the tested benign conditions. They do not establish temperature qualification, vibration immunity, environmental invariance, or adversarial robustness.

### 5.5. Embedded Performance

The follow-up acquisition-plus-extraction batch means were 1414.2877, 1414.2867, and 1414.2876 microseconds per 18-bit cycle, giving a mean of 1414.28733 microseconds and a sample standard deviation of 0.00055 microseconds across batch means. This small dispersion does not quantify clock accuracy or individual-cycle jitter. Acquisition-only means were 1414.2872, 1414.2868, and 1414.2869 microseconds. The extraction-loop means were 0.250949, 0.250942, and 0.250942 microseconds per iteration (mean 0.250944 microseconds). No sensor-read errors were reported. The nominal acquired-bit rate is 12.7273 kbit/s, excluding serial formatting/transmission; it is not an entropy-rate estimate. Sequential multiplexer selection and I2C reads dominate this workload.

### 5.6. Discussion

The historical V1 result shows that von Neumann processing substantially improved marginal balance, but the discovered fixed-position structure also demonstrates why balance and Shannon entropy are insufficient. The revised V2 design explicitly reads Gyro X/Y/Z, preserves all 18 bit positions, and shows no recurrence of the V1 fixed-position defect in the evaluated datasets.

Post hoc inspection of the historical V1 firmware and stream showed a repeating per-sensor structure in which eight of twelve extracted positions were deterministically zero and only four positions varied. The approximately 83/17 aggregate imbalance therefore arose primarily from the acquisition/bit-selection method rather than from a demonstrated physical entropy distribution of the MEMS devices. V2 removes this artifact by reading Gyro X/Y/Z explicitly and retaining two LSBs from every axis. Because the V1 samples were not independent and identically distributed, the von Neumann result is retained only as a conditioned-output case study and is not used to claim source entropy.

The prototype separates cloud-generated initialization material from local acquisition, but it does not establish a quantum root of trust. The embedded seed constant, Xorshift128+, and XOR telemetry masking are laboratory mechanisms. Production security would require authenticated per-device provisioning, protected storage, standard key derivation, a vetted DRBG, and authenticated encryption.

A deterministic generator expands existing state but does not create entropy. The measured ESP32 esp_random() interface produced approximately 57.9198 Mbit/s, whereas the three-sensor V2 acquisition produced 12.7273 kbit/s. EntroMEMS is therefore not presented as a faster replacement for the platform RNG; its defensible role is an experimental, physically distinct auxiliary input. The documented ESP32 RNG operating conditions must also be observed because continuous physical entropy depends on the enabled RF or SAR entropy source. The operating-state qualification follows the official ESP-IDF RNG documentation [28]. In the follow-up benchmark, Wi-Fi and Bluetooth were not started by the sketch; the timing result does not validate the entropy quality of the RNG output.

The follow-up benchmark build reported 327,296 bytes free from a 375,440-byte heap and a 296,704-byte firmware image on a 4,194,304-byte flash device. The esp_random() batch means were 0.552491, 0.552485, and 0.552488 microseconds per call (overall mean 0.552488 microseconds), including loop and accumulation overhead. Memory figures apply to this instrumented build. Calibrated energy, task-level CPU utilization, and end-to-end quantum-provisioning latency were not measured and are not estimated.

Proposed health monitoring. A deployment-oriented design would run startup and continuous tests on raw samples before conditioning. At minimum, the binary source would require NIST SP 800-90B Repetition Count [27] and Adaptive Proportion tests with thresholds derived from a validated entropy estimate and an explicit false-positive target. Sensor communication errors, malformed records, saturation, unchanged multi-axis words, missing channels, and persistent cross-channel correlation would trigger additional device-specific checks. On failure, the implementation would discard affected samples, suppress entropy credit and DRBG reseeding, preserve the prior DRBG state, record a diagnostic event, and enter a defined degraded or fail-safe mode. These mechanisms are proposed and were not implemented in the present firmware.

Physical attack surface. MEMS output can be influenced by mechanical coupling, vibration, acoustic excitation, temperature, electromagnetic interference, supply disturbance, and direct manipulation of the sensor or I2C path. The benign perturbation experiments in this study do not model a capable adversary. A production design would require shielding and mounting analysis, sensor plausibility and cross-sensor consistency checks, rate limits on entropy credit, and adversarial testing across the declared operating envelope.

The study uses one ESP32 board and three sensor units. The environmental experiments were benign and not calibrated qualification tests: sensor temperature was not measured directly, external heating may also have introduced airflow-induced mechanical effects, and adversarial injection was not evaluated. Full SP 800-90B validation is not claimed because the prescribed 1000 × 1000 restart dataset, complete physical noise-source model, restart assessment, and continuous health-test design remain outstanding.

Overall, the results support feasibility and reproducible characterization of a proof-of-concept auxiliary entropy path. They do not demonstrate a complete production security architecture, a validated entropy source, or fleet-scale provisioning.

## 6. Conclusions

This paper presented EntroMEMS as a proof-of-concept architecture combining quantum-derived initialization material with structured MEMS gyroscope acquisition on an ESP32. The revision corrects the sample model, evaluates sensors separately, and adds an official conditioned-output single-sequence assessment, dependency-aware sequential estimation, robustness experiments, and performance measurements.

The historical conditioned sequence was nearly balanced, with P(0) = 0.500111, P(1) = 0.499889, and a Shannon entropy of 0.9999999644 bit/symbol. In the official NIST SP 800-22 STS 2.1.2 single-sequence assessment, all 188 individual *p*-values were at least 0.01. These output diagnostics are complemented by the lower dependency-aware non-IID estimates obtained from raw V2 samples; neither result is presented as full entropy-source validation or proof of cryptographic suitability.

The prototype demonstrates technical integration, not secure provisioning. A fleet deployment should use unique per-device seed material, authenticated delivery, protected storage, inventory, revocation, and reprovisioning; reuse of a common fleet-wide seed is rejected.

Future work will collect the prescribed restart dataset, define and validate continuous health tests, measure power using calibrated instrumentation and measure provisioning latency, evaluate additional devices under calibrated environmental conditions, and replace the experimental generator and masking mechanism with a vetted DRBG and authenticated encryption. Local reseeding will be performed only through the DRBG reseed interface after health testing, conditioning, and conservative entropy accounting.

EntroMEMS should therefore be interpreted as a reproducible proof of concept and measurement study. Its results motivate further entropy-source validation and secure-system engineering but do not by themselves establish cryptographic suitability or a protected root of trust.

## Figures and Tables

**Table 1:** Hardware and software resources used in the proposed EntroMEMS framework.

Category	Resource	Purpose
Embedded controller	ESP32 DevKit (Shenzhen Xunhuawei Trading Electronics Co., Ltd., Shenzhen, Guangdong, China)	Acquisition and embedded benchmark
MEMS sensors	3 x MPU-9250/MPU-6500-class ((Shenzhen Hao An Da Technology Co., Ltd., Shenzhen, China)	Separate Gyro X/Y/Z candidate samples
I2C multiplexer	TCA9548A ((Shenzhen Baishun Ming Core Electronics Co., Ltd., Shenzhen, China)	Sequential sensor selection
Host computer	Windows 11 workstation	Capture and offline analysis
Embedded software	Arduino framework	V2 acquisition firmware
Data processing	Python 3	Parsing, conditioning, descriptive statistics
Quantum SDK	Qiskit Runtime	IBM Quantum experiment
Statistical testing	NIST SP 800-22 Rev. 1a	Conditioned-output diagnostics
Entropy estimation	NIST SP 800-90B tool v1.1.8	Non-IID sequential assessment

**Table 2:** Measured prototype throughput and conceptual roles of randomness sources and generators.

Approach	Physical Input	Evidence and Role	Unimplemented Requirements or Limitations
Deterministic generator	No new physical entropy	Conceptual comparison; expands state. Xorshift128+ is the prototype baseline, not a benchmarked secure DRBG.	A vetted DRBG and protected seed/state are required for production.
ESP32 esp_random()	Hardware-state dependent	Measured API throughput: 57.9198 Mbit/s, including loop overhead.	Timing does not validate entropy quality; documented hardware conditions apply.
MEMS V2 acquisition	Candidate sensor variation	Measured acquisition plus extraction: 12.7273 kbit/s. Auxiliary-entropy role is proposed.	Not a validated entropy rate or standalone secure generator; health tests and secure reseeding are not implemented.
Cloud quantum-derived material	External initialization event	Retained 1024-bit pool; first 128 bits load the prototype state. No provisioning-rate benchmark.	Authenticated provisioning, protected storage, and lifecycle controls are proposed.

**Table 3:** Statistical properties of the conditioned bitstream.

Metric	Historical V1 Raw Stream	Conditioned Evaluation Sequence
Sequence length	12,500,028 bits	1,000,000 bits
P(0)	approximately 0.83	0.500111
P(1)	approximately 0.17	0.499889
Shannon entropy	0.6500918889 bit/symbol	0.9999999644 bit/symbol
Processing state	Unconditioned historical acquisition	Von Neumann conditioned

**Table 4:** NIST SP 800-22 STS 2.1.2 individual results for one 1,000,000-bit conditioned MEMS-derived sequence.

NIST SP 800-22 Test Category	Individual *p*-Value Result(s)	Single-Sequence Interpretation
Frequency (Monobit)	1/1; *p* = 0.824314	Did not reject at alpha = 0.01
Frequency within a Block	1/1; *p* = 0.967420	Did not reject at alpha = 0.01
Runs	1/1; *p* = 0.191569	Did not reject at alpha = 0.01
Longest Run of Ones	1/1; *p* = 0.335446	Did not reject at alpha = 0.01
Binary Matrix Rank	1/1; *p* = 0.511145	Did not reject at alpha = 0.01
Discrete Fourier Transform	1/1; *p* = 0.419353	Did not reject at alpha = 0.01
Non-overlapping Template Matching	148/148; 0.010783–0.999223	No applicable template rejected
Overlapping Template Matching	1/1; *p* = 0.105978	Did not reject at alpha = 0.01
Maurer’s Universal	1/1; *p* = 0.177209	Did not reject at alpha = 0.01
Linear Complexity	1/1; *p* = 0.844284	Did not reject at alpha = 0.01
Serial	2/2; 0.039707–0.178275	Neither component rejected
Approximate Entropy	1/1; *p* = 0.881633	Did not reject at alpha = 0.01
Cumulative Sums	2/2; 0.945889–0.999360	Neither direction rejected
Random Excursions	8/8; 0.237554–0.872610	No applicable state rejected
Random Excursions Variant	18/18; 0.142487–0.975875	No applicable state rejected

## Data Availability

The datasets, firmware, analysis scripts, NIST inputs and outputs, and follow-up measurement records are provided in the Supplementary Materials. The retained quantum job identifier and screenshots are included, but the full machine-readable job metadata, calibration snapshot, executed transpiled circuit, and exact historical Qiskit versions are unavailable. Historical V2 configuration/per-read timing logs and the individual original benchmark reports were not recovered. These limitations are distinguished from the newly documented follow-up measurements.

## Supplementary Materials

The following supporting information can be downloaded at: https://www.mdpi.com/article/10.3390/s26206494/s1, EntroMEMS_Supplementary_Materials_v2 contains the original V2/NIST evidence, retained V1 raw data and conditioning code, a verified conditioning reproduction procedure, redacted quantum generation source and loading firmware, quantum provenance artifacts, and the 7 October 2026 configuration, cadence, and benchmark sketches and logs. REPRODUCTION.md maps procedures to files and identifies unavailable historical artifacts; MANIFEST.csv records file sizes and SHA-256 hashes.
